# Using selection index theory to estimate consistency of multi-locus linkage disequilibrium across populations

**DOI:** 10.1186/s12863-015-0252-6

**Published:** 2015-07-19

**Authors:** Yvonne C.J. Wientjes, Roel F. Veerkamp, Mario P.L. Calus

**Affiliations:** Animal Breeding and Genomics Centre, Wageningen UR Livestock Research, 6700 AH Wageningen, The Netherlands; Animal Breeding and Genomics Centre, Wageningen University, 6700 AH Wageningen, The Netherlands

**Keywords:** Multi-locus LD, Consistency of LD, Genomic prediction, Across population genomic prediction, Accuracy, Selection index theory

## Abstract

**Background:**

The potential of combining multiple populations in genomic prediction is depending on the consistency of linkage disequilibrium (LD) between SNPs and QTL across populations. We investigated consistency of multi-locus LD across populations using selection index theory and investigated the relationship between consistency of multi-locus LD and accuracy of genomic prediction across different simulated scenarios. In the selection index, QTL genotypes were considered as breeding goal traits and SNP genotypes as index traits, based on LD among SNPs and between SNPs and QTL. The consistency of multi-locus LD across populations was computed as the accuracy of predicting QTL genotypes in selection candidates using a selection index derived in the reference population. Different scenarios of within and across population genomic prediction were evaluated, using all SNPs or only the four neighboring SNPs of a simulated QTL. Phenotypes were simulated using different numbers of QTL underlying the trait. The relationship between the calculated consistency of multi-locus LD and accuracy of genomic prediction using a GBLUP type of model was investigated.

**Results:**

The accuracy of predicting QTL genotypes, i.e. the measure describing consistency of multi-locus LD, was much lower for across population scenarios compared to within population scenarios, and was lower when QTL had a low MAF compared to QTL randomly selected from the SNPs. Consistency of multi-locus LD was highly correlated with the realized accuracy of genomic prediction across different scenarios and the correlation was higher when QTL were weighted according to their effects in the selection index instead of weighting QTL equally. By only considering neighboring SNPs of QTL, accuracy of predicting QTL genotypes within population decreased, but it substantially increased the accuracy across populations.

**Conclusions:**

Consistency of multi-locus LD across populations is a characteristic of the properties of the QTL in the investigated populations and can provide more insight in underlying reasons for a low empirical accuracy of across population genomic prediction. By focusing in genomic prediction models only on neighboring SNPs of QTL, multi-locus LD is more consistent across populations since only short-range LD is considered, and accuracy of predicting QTL genotypes of individuals from another population is increased.

**Electronic supplementary material:**

The online version of this article (doi:10.1186/s12863-015-0252-6) contains supplementary material, which is available to authorized users.

## Background

In genomic prediction, marker information is used to predict breeding values for selection candidates based on estimated marker effects in a reference population consisting of individuals with phenotypes and marker genotypes. The accuracy of predicting genomic breeding values depends on the size of the reference population, the heritability of the trait, and on the level of family relationships between the reference population and selection candidates, e.g. [1–3]. Moreover, the accuracy is influenced by the level of linkage disequilibrium (LD), i.e. non-random associations, between the single nucleotide polymorphism (SNP) markers and quantitative trait loci (QTL) influencing the trait of interest [4]. The higher the level of LD, the more accurate breeding values can be predicted for the selection candidates [5]. Therefore, the consistency of linkage phase between SNPs and QTL across populations has been suggested to be an important factor determining the success of across and multi population genomic prediction [6, 7]. Within a population, the level of LD between a QTL and a SNP depends on the effective population size, the recombination rate, the distance between the QTL and SNP on the genome, and the difference in allele frequency between the QTL and SNP [8]. Several studies showed different LD patterns across different cattle [9, 10], chicken [11, 12], pig [13] and human [14] populations. In different livestock species, however, the consistency of linkage phase across populations is found to be reasonable high at short distances on the genome [9, 12, 15], and depending on the degree of relatedness between the populations; the higher the relatedness between the populations, the higher the consistency of LD [12].

The studies investigating the consistency of LD across populations focused on the LD between two loci. However, genomic prediction models trained within populations are expected to use more than one SNP to capture the genetic variance explained by one QTL [16]. Hayes *et al.* [17] for example showed a substantial increase in the proportion of the QTL variance captured by the SNPs when going from haplotypes based on 2 SNPs per haplotype to 4 SNPs per haplotype and from 4 SNPs per haplotype to 6 SNPs per haplotype. Moreover, the proportion of the QTL variance explained by haplotypes with more than 2 SNPs was higher than the proportion that could be explained by the SNP in highest LD with the QTL [17]. Also for fine mapping QTL, the use of haplotypes consisting of multiple SNPs is shown to be beneficial compared to using one SNP at a time [18–20]. This indicates that SNPs in less strong LD with the QTL might be helpful in genomic prediction, and linear combinations of several linked SNPs form the within population prediction equation. Therefore, a measure of multi-locus LD, compared to the average LD between two adjacent loci, might be better able to explain the contribution of LD to the accuracy of genomic prediction. This might especially be important for situations with multiple populations, because the consistency of LD across populations is decreasing more rapidly at increasing distances on the genome [9, 21, 10].

The first objective of this study was to investigate the consistency of multi-locus LD across different populations using selection index theory. The consistency of multi-locus LD is one of the components of the accuracy of genomic prediction, therefore, the second objective was to investigate the relationship between consistency of multi-locus LD and accuracy of genomic prediction across different simulated within and across population genomic prediction scenarios. Three different cattle breeds with real SNP genotype information were used to represent different populations. Phenotypes of the individuals were simulated by sampling QTL from the SNPs, such that the actual QTL genotypes influencing the phenotypes were known.

## Methods

### Prediction accuracies

#### Using selection index theory to predict QTL genotypes

In this study, the consistency of multi-locus LD across different populations is investigated using selection index theory [22–24], which is equivalent to multiple regression of the QTL genotypes on the SNP genotypes. In the selection index calculations, a regression equation to predict the QTL genotypes (i.e. the breeding goal traits) using SNP genotypes (i.e. the index traits) was derived in population *A* and the accuracy of this equation to predict the QTL genotypes in population *B* was investigated. This approach is different from other studies investigating the consistency of LD across populations, e.g. [9, 10, 15], where the consistency of LD was calculated using the correlation of the LD measure *r* between two single loci across populations. The advantage of our selection index method is that a measure is obtained of explaining the QTL genotypes using the information of multiple SNPs instead of a single SNP.

In population *A*, a selection index can be derived to predict the QTL genotype for a single individual using all SNP genotypes of that same individual, following:

1 $$ {I}_i={\mathbf{b}}_A\hbox{'}{\mathbf{x}}_i $$

in which *I*_*i*_ forms the selection index for individual *i*, **b**_*A*_ is a vector containing regression coefficients on the SNP genotypes to predict *I*_*i*_, and **x**_*i*_ is a vector containing all SNP genotypes of individual *i*.

Rather than predicting *I*_*i*_, the aim is to predict the aggregated genotype including all QTL:

2 $$ {H}_i=\mathbf{v}\hbox{'}{\mathbf{g}}_i $$

in which *H*_*i*_ is the aggregate genotype of individual *i*, **v** is a vector with weighting factors for each of the QTL genotypes and **g**_*i*_ is a vector containing the genotype for each QTL of individual *i*.

The regression coefficients on the SNP genotypes that would optimize the prediction accuracy of *H* can be calculated as [25]:

3 $$ {\mathbf{b}}_A={\mathbf{P}}_A^{-1}{\mathbf{G}}_A\mathbf{v} $$

in which **P**_*A*_ is the covariance matrix (based on LD) between all SNPs in population *A* and **G**_*A*_ is the covariance matrix between SNPs and QTL in population *A*. Then the prediction accuracy of predicting the QTL genotype in another population, i.e. population *B*, using **b**_*A*_ can be calculated as [26]:

4 $$ {r}_{IH}=\frac{{\mathbf{b}}_A\hbox{'}{\mathbf{G}}_B\mathbf{v}}{\sqrt{{\mathbf{b}}_A\hbox{'}{\mathbf{P}}_B{\mathbf{b}}_A\mathbf{v}\hbox{'}{\mathbf{C}}_B\mathbf{v}}} $$

in which **G**_*B*_ is the covariance matrix between SNPs and QTL in population *B*, **P**_*B*_ is the covariance matrix of SNPs in population *B* and **C**_*B*_ is the covariance matrix of QTL in population *B*.

#### Using a genomic best linear unbiased prediction model to estimate breeding values

To investigate the relationship between the prediction accuracies of the QTL genotypes and the accuracies of predicting genomic breeding values, the following genomic-relationship-matrix residual maximum likelihood (GREML) model was used:

5 $$ \mathbf{y}=\mathbf{X}\mathbf{b}+\mathbf{Zg}+\mathbf{e} $$

in which **y** is a vector containing phenotypes, **b** is a vector containing fixed effects, **X** is an incidence matrix that allocates the fixed effects to the individuals, **g** is a vector containing the predicted genomic breeding values ~ *N*(0,**GRM**σ_*g*_^2^), **GRM** is a genomic relationship matrix based on SNPs (calculation of **GRM** is explained later), **Z** is an incidence matrix that allocates the genomic breeding values to the individuals and **e** is a vector containing the residuals ~ *N*(0,**I**σ_*e*_^2^). The GREML model is equivalent to the commonly known genomic best linear unbiased prediction (GBLUP) model, except that it estimates the variances using residual maximum likelihood (REML) instead of assuming that the variances are known.

### Simulations to investigate the prediction accuracies

#### Genotypes

Genotypes of 1285 dairy cows from the Netherlands were used, originating from three different breeds (1033 Holstein Friesians (HF), 105 Groninger White Headed (GWH), and 147 Meuse-Rhine-Yssel (MRY)). The genotypes of MRY and GWH animals were obtained by isolating DNA from whole blood samples of the animals. Blood samples were collected in accordance with the guidelines for the care and use of animals as approved by the ethical committee on animal experiments of ID-LELYSTAD (protocol: 2011062). No approval was obtained for the HF genotypes, because these genotypes were obtained from an existing database.

All animals originated for at least 87.5 % from one of the three breeds, so were considered to be pure-bred animals. The HF animals were genotyped with the Illumina BovineSNP50 Beadchip (50 k, Illumina, San Diego, CA), and genotypes were imputed to high density (777 k) using 3150 HF animals in the reference population as described in Pryce *et al.* [27]. The GWH and MRY animals were genotyped with the Illumina BovineHD Beadchip (777 k, Illumina, San Diego, CA). The quality checks and the criteria for including the SNP genotypes in the combined dataset of the three breeds are described in Wientjes *et al*. [28]. For each of the individuals, both genotype (coded as 0, 1 and 2) and phased allele information (coded as 0 and 1) was available. Phasing of the allele genotypes was done using the software package Beagle [29]. From those high density genotypes, arbitrarily the SNP genotypes of three chromosomes (*Bos Taurus* chromosome (BTA) 13, BTA 23 and BTA 28) were selected to reduce computation time and to increase the power of the study to estimate breeding values. The three selected chromosomes contained 31 503 SNPs, which was about 10 % of the SNPs from the entire combined dataset. The characteristics of the 31 503 SNPs used in this study are shown in Table 1.

**Table 1:** Characteristics of the SNPs in each of the different breeds

Characteristics of the SNPs	HF^1^	GWH^2^	MRY^3^
Number of segregating SNPs	31 483	30 449	31 262
Number of breed-specific SNPs	14	6	3
Average MAF^4^ of all SNPs	0.279	0.251	0.266
Average MAF^4^ of segregating SNPs	0.279	0.260	0.268
Number of SNPs with MAF^4^ ≤ 0.1	4266	6530	5308
Number of SNPs with 0.1 < MAF^4^ ≤ 0.2	5587	5803	5609
Number of SNPs with 0.2 < MAF^4^ ≤ 0.3	6558	5745	6623
Number of SNPs with 0.3 < MAF^4^ ≤ 0.4	7430	6718	6657
Number of SNPs with 0.4 < MAF^4^ ≤ 0.5	7662	6707	7306

^1^ *HF* Holstein Friesian

^2^ *MRY* Meuse-Rhine-Yssel

^3^ *GWH* Groninger White Headed

^4^ *MAF* Minor allele frequency

From all 31 503 SNPs, randomly 5000 SNPs were selected to become candidate QTL from which the actual QTL were sampled. The other 26 503 SNPs were used as SNP markers in this study. With this approach, it was possible to randomly sample QTL from the candidate QTL in each of the replicates, while keeping the set of SNP markers constant across the replicates to reduce the computational demands. To limit the number of possible singularities in the matrices needed for the selection index calculations, SNPs with a correlation above 0.85 or below −0.85 with another SNP on the same chromosome were deleted, irrespective of their allele frequency. Moreover, SNPs that were not segregating in one of the breeds were deleted as well. Deleting those SNPs reduced the total number of SNPs from 26 503 to 4541, of which 1655 SNPs were located on BTA 13, 1515 on BTA 23, and 1371 on BTA 28.

#### Phenotypes

Phenotypes were simulated for each individual by randomly sampling 3000, 300, 30, or 3 QTL from the group of 5000 candidate QTL and by sampling their allele substitution effects from *N*(0,1), using the same effects for each of the breeds. An additive model, without considering epistatic interactions or dominance effects, was assumed. The simulated allele substitution effects were multiplied with the QTL genotypes, coded as 0, 1 and 2, to calculate a true breeding value (TBV) for each of the individuals. Those TBVs were rescaled to a mean of 0 and a variance of 1 across breeds for all of the scenarios. Thus, when the number of QTL underlying the trait was lower, each QTL explained a larger part of the genetic variance. For each individual, an environmental effect was sampled from *N*(0,$\left(\frac{1}{h^2}-1\right)$*variance of TBV corrected for mean TBV within breed), in which *h*^2^ is the heritability of the simulated trait. This approach enables to sample the environmental term from the same distribution for each individual, independent of the breed, and to keep the heritability more or less constant across the breeds [28]. The phenotype for each individual was calculated as the sum of its TBV and its randomly sampled environmental effect. Please note that the TBVs were only corrected for the mean TBV to calculate the environmental variance, the TBVs and the phenotypes still contained the breed effect.

Two different heritabilities were used to simulate phenotypes, namely 0.3 and 0.95. The same subsets of QTL were used to simulate phenotypes for the two heritabilities, but allele substitution effects and environmental effects were different. For all scenarios, simulations were replicated 100 times for each scenario. A more detailed description of the simulations of phenotypes can be found in Wientjes *et al.* [28].

In general, QTL underlying complex traits are expected to have a lower minor allele frequency (MAF) than the SNPs, due to ascertainment bias of the SNPs on the chip [30, 31]. To investigate if selecting QTL randomly from the SNPs could affect our results, phenotypes were also simulated by selecting QTL from the 5000 candidate QTL with an average MAF across the breeds below 0.1. The average MAF across the breeds was calculated by giving an equal weight to each of the three breeds, indicating that the allele frequency in each of the breeds ranged between 0 and 0.3, resulting in sampling QTL from 480 candidate QTL. Simulating phenotypes by selecting QTL with a low MAF was only done using 3 QTL underlying the trait and a heritability of 0.95 using 100 replicates.

#### Scenarios

The consistency of multi-locus LD and accuracy of genomic prediction were evaluated in five different scenarios (Table 2). In the base scenario, within population genomic prediction was applied, using HF individuals both in the reference population and as selection candidates. The other four scenarios used across population genomic prediction, indicating that the population of the selection candidates (GWH or MRY) was not included in the reference population, and that all individuals of the predicted population were used for the validation. To perform validation in the within population scenario, 10-fold cross validation was used in which the individuals were randomly divided in 10 equally sized groups using each group once as selection candidates and the other groups as reference population. In each replicate, the division of the individuals over the groups was the same.

**Table 2:** Overview of the breeds used in the different reference populations and as selection candidates

	Reference population		Predicted individuals	
Scenario	Breed(s)	Number of individuals	Breed	Number of individuals
Base	HF^1^	928-929	HF^1^	103-104
1	HF^1^	1033	GWH^3^	105
2	HF^1^ + MRY^2^	1180	GWH^3^	105
3	HF^1^	1033	MRY^2^	147
4	HF^1^ + GWH^3^	1138	MRY^2^	147

^1^ *HF* Holstein Friesian

^2^ *MRY* Meuse-Rhine-Yssel

^3^ *GWH* Groninger White Headed

#### Selection index calculations

The selection index calculations were performed for each scenario by defining a selection index to predict QTL genotypes in the reference population (Equation 3) and to calculate the prediction accuracy of this selection index in the selection candidates (Equation 4). In the **P-**, **G-**, and **C-**matrices (Equation 3 and 4), we used the correlations between SNPs and QTL that were calculated based on the phased alleles of SNPs and QTL of all individuals in either the reference population or the group of selection candidates. By using correlations instead of covariances, each SNP explains an equal amount of the genetic variance, similar to the commonly used assumption in GREML. Moreover, the square of the correlation between phased alleles at two loci, *r*^2^, is commonly used as a measure for LD between loci [8].

Across the different replicates, the subset of SNPs was constant, as indicated previously. This indicates that the **P-**matrices within both the reference population and the selection candidates were constant across the replicates. The set of QTL differed for each replicate, so both the **G-** and **C-**matrices were specific for each of the replicates. Correlations among SNPs and QTL and between SNPs and QTL on different chromosomes were taken into account as well to make the analyses consistent with the GREML analyses that did not differentiate between the chromosomes. To prevent problems due to non-positive definiteness of the final matrices, the **P-** and **C-**matrices were bended following the unweighted bending procedure described by Jorjani *et al.* [32] by setting the eigenvalues of the matrix lower than 10e^−6^ to 10e^−6^.

Two different weightings of the QTL in the overall breeding goal, vector **v** in Equation 2, 3 and 4, were used; either QTL were weighted equally (**v** is a vector of ones), or each QTL was weighted based on its simulated allele substitution effect to take into account that it is more important to accurately predict the QTL genotype of QTL with large effects than for QTL with small effects. Weighting the QTL based on their allele substitution effects was only performed for the phenotypes simulated using a heritability of 0.95, both when QTL were randomly selected and when QTL were selected with a low MAF.

In the analyses described above, all SNPs across the whole genome were taken into account to explain the QTL genotypes. The SNPs more closely located to a QTL are supposed to have a higher and more consistent LD with the QTL across populations, e.g. [9, 12, 15]. To investigate if the accuracy of predicting QTL genotypes would be increased when focusing only on the SNPs surrounding a QTL, the analyses with 3 randomly selected QTL underlying the trait were repeated using only the four surrounding SNPs (two at either side) of each QTL. When the number of SNPs from one side of the QTL was insufficient, i.e. when the QTL was located at the end of a chromosome, more SNPs from the other side of the QTL were added to obtain four SNPs per QTL. Those analyses were only performed by using an equal weight of the QTL in the overall breeding goal.

#### Estimating breeding values using GREML

To estimate breeding values for the individuals, the GREML model (Equation 5) was run in ASReml [33], including breed as the only fixed effect. The **GRM** matrix that was used in the model was calculated as $\mathbf{G}\mathbf{R}\mathbf{M}=\frac{\mathbf{XX}\hbox{'}}{n}$ [34, 35], in which *n* represents the number of SNP markers (*n* = 4541) and the **X-**matrix contains standardized genotypes, calculated as ${x}_{ij}=\frac{g_{ij}-2{p}_j}{\sqrt{2{p}_j\left(1-{p}_j\right)}}$, in which *g*_*ij*_ codes the genotype for individual *i* at marker locus *j* as 0, 1 and 2, and *p*_*j*_ is the allele frequency at marker locus *j* for the second allele (for which the homozygote genotype is coded 2) averaged over the three breeds. After adjusting the inbreeding level in **GRM** to the inbreeding level in the pedigree based relationship matrix **A**, the **GRM** matrix was regressed back to the **A** matrix to reduce the effect of sampling the SNPs on the chip. For each of the scenarios, a different **GRM** matrix was calculated, containing only the individuals included in that scenario. For a more detailed description of calculating **GRM**, see Wientjes *et al.* [28].

For each population, the accuracy of genomic prediction was calculated as the correlation between the estimated breeding values and the simulated TBVs. Averages and standard errors of the accuracies of genomic prediction were calculated across replicates.

## Results

### Regression coefficients

**Fig. 1:**
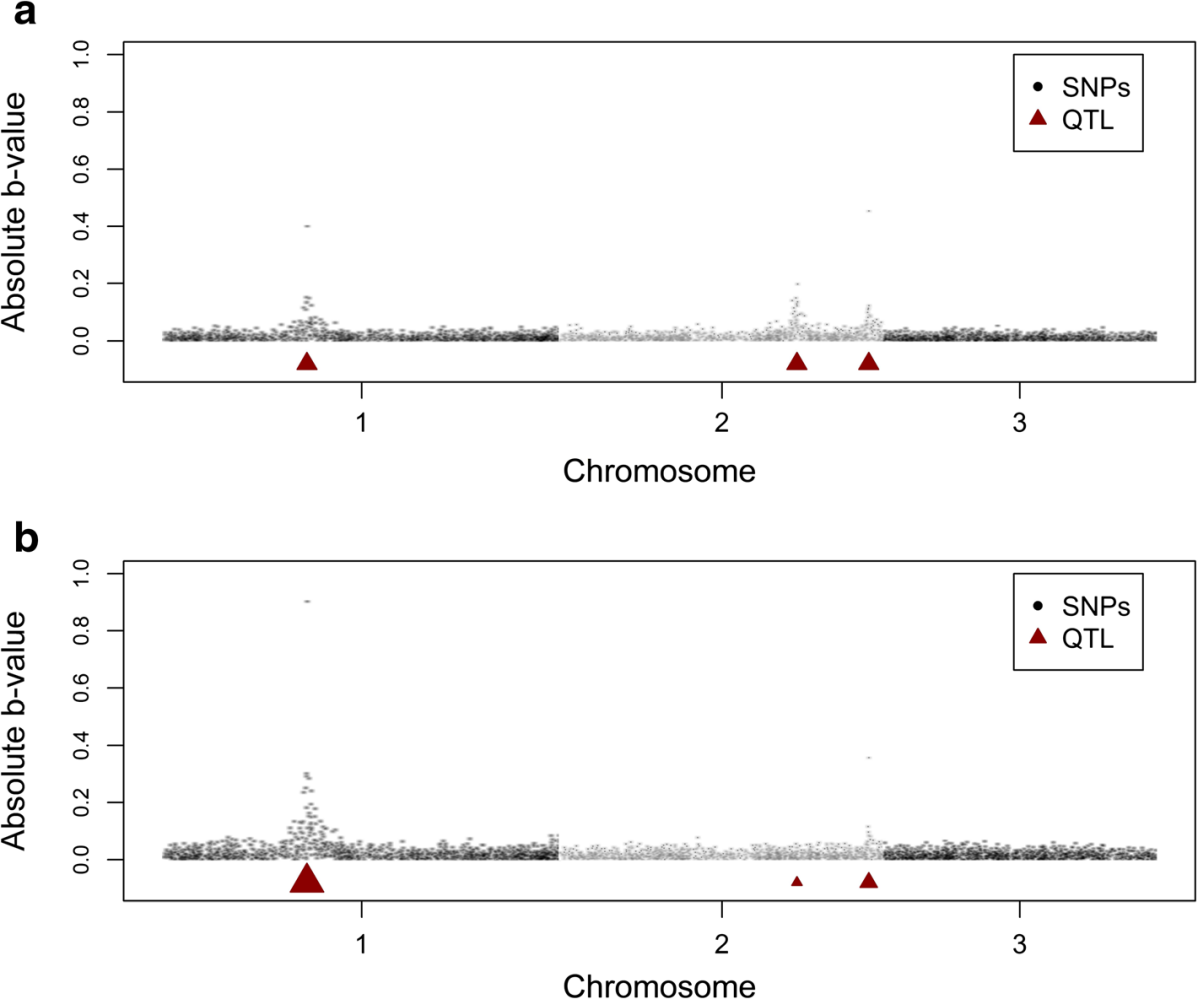
Absolute estimated regression coefficients (**b**-values) for each SNP to predict the QTL genotypes of 3 randomly selected QTL. Absolute regression coefficients for each of the SNPs estimated in a Holstein Friesian reference population (**b**_*RP*_) to predict the QTL genotypes of 3 randomly selected QTL with (**a**) equal weight for each of the QTL, or (**b**) QTL weighted differently, based on their allele substitution effects, in the overall breeding goal. The size of the triangle represents the weight of the QTL in the overall breeding goal of the selection index calculations, i.e. the allele substitution effect in (**b**)

The regression coefficients on the SNP genotypes to predict the QTL genotypes derived in the Holstein Friesian reference population using selection index calculations (Equation 2; **b**_*RP*_) are presented in Fig. 1 for one of the replicates with 3 randomly selected QTL underlying the trait. This figure clearly shows that the SNPs surrounding a QTL were given a higher weight to predict the QTL genotypes, due to the greater correlations between those SNPs and the QTL. When QTL were weighted based on their different allele substitution effects, mainly the SNPs surrounding the QTL with a large effect were given a higher weight. The same patterns were also seen when the number of QTL was higher, although the pattern was less clear due to the higher number of QTL (See Additional file 1: Figures S1-S3), and when the MAF of QTL was lower (See Additional file 2: Figure S4).

### Accuracy of predicting QTL genotypes using selection index theory

**Fig. 2:**
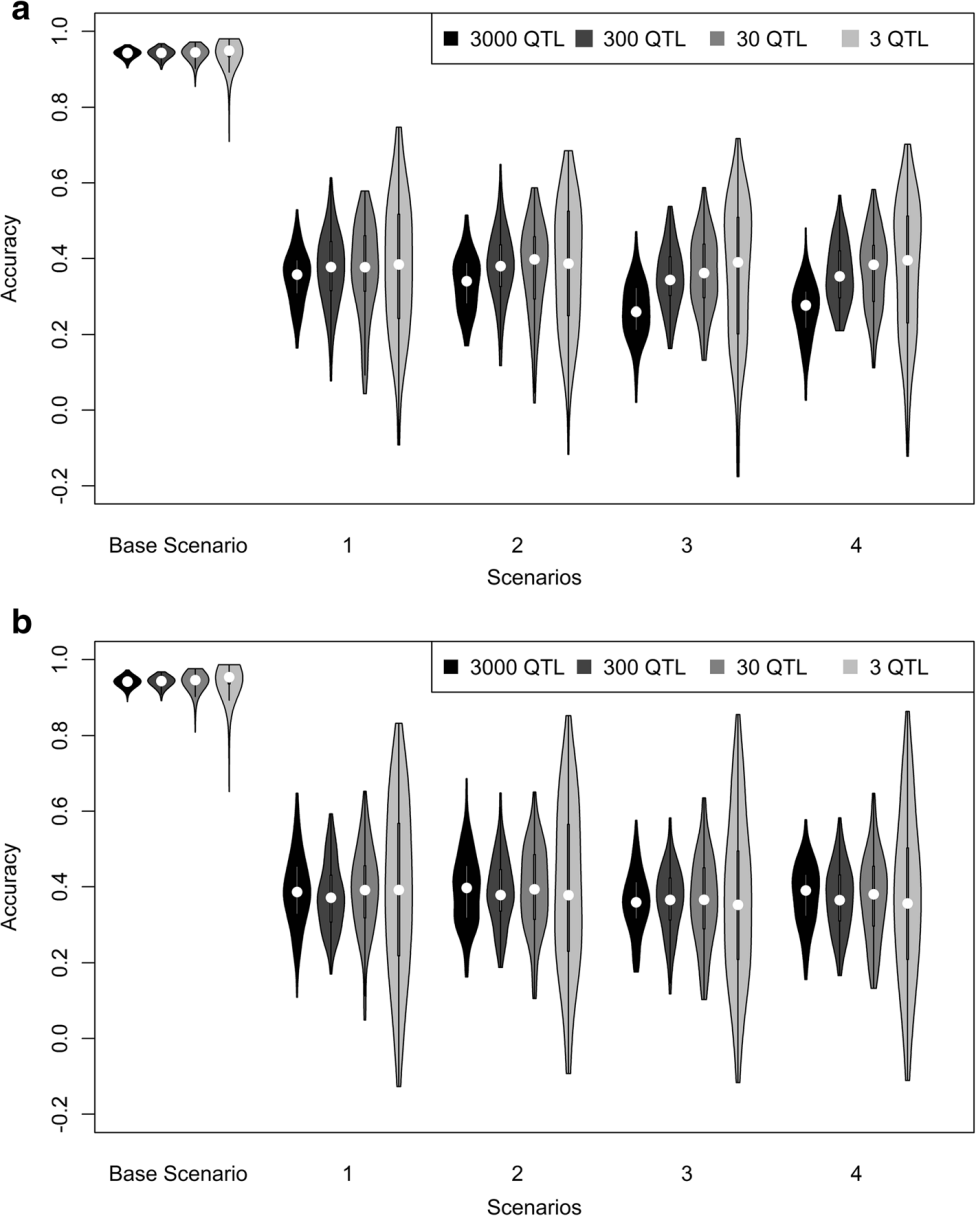
Accuracies of predicing genotypes of randomly sampled QTL using selection index theory. Violin plot depicting the accuracies of selection index theory to predict the QTL genotypes of randomly sampled QTL using (**a**) equal weight for each of the QTL, or (**b**) QTL weighted differently, based on their allele substitution effects, in the overall breeding goal for five different scenarios. Base = reference population Holstein Friesian (HF), selection candidates HF; 1 = reference population HF, selection candidates Groninger White Headed (GWH); 2 = reference population HF and Meuse-Rhine-Yssel (MRY), selection candidates GWH; 3 = reference population HF, selection candidates MRY; 4 = reference population HF and GWH, selection candidates MRY

Accuracies of predicting the QTL genotypes for the selection candidates, using a selection index derived in the reference population based on all SNPs, are shown in Fig. 2 when QTL were randomly sampled. Since this prediction accuracy is a measure of the consistency of multi-locus LD (MLLD) between the selection candidates and the reference population, hereafter this accuracy will be referred to as acc_MLLD. In the within population scenarios, average acc_MLLD was around 0.94. As expected, average acc_MLLD was much lower for the across population scenarios due to differences in LD across populations with an average acc_MLLD of ~0.37 for GWH and ~0.34 for MRY using HF as reference population. Adding another population to the HF reference population did not affect the prediction accuracy.

The average acc_MLLD seems to be independent from the number of QTL underlying the trait for the within as well as for the across population scenarios, both when QTL had an equal weight and when QTL were weighted based on their allele substitution effects. Only when 3000 QTL were underlying the trait and QTL had an equal weight in the breeding goal, acc_MLLD was slightly lower compared to the across population scenarios with fewer QTL. Standard errors were in general very small, but tended to be slightly larger for the scenarios with a lower number of QTL.

Weighting the QTL equally or based on their allele substitution effects resulted in similar values for acc_MLLD, both for the within and across population scenarios. This was also expected beforehand, since the consistency of multi-locus LD across populations was supposed to be a characteristic of the investigated populations. Giving different weights to the QTL only resulted in giving more emphasis on predicting QTL with a large effect, but it had no effect on the LD structure of that QTL with the surrounding SNPs. The only exception to this pattern was again the across population scenario with 3000 QTL underlying the trait, where acc_MLLD was higher when QTL were weighted differently compared to weighting the QTL equally.

By focusing only on the four SNPs surrounding a QTL, the accuracy of predicting the QTL genotypes of the selection candidates decreased by 19 % for the within population scenario (Table 3). For the across population scenarios, however, the prediction accuracy increased by approximately 53 % (Table 3). As a consequence, the difference in prediction accuracy of the QTL genotypes between the within and across population scenarios was substantially reduced compared to the analyses using all SNPs.

**Table 3:** Average prediction accuracies of QTL genotypes using all SNPs or only the neighboring SNPs of the QTL. The results are for different within and across population scenarios with 3 QTL underlying the trait and with an equal weight of the QTL in the overall breeding goal

	Reference population	Selection candidates	Average prediction accuracy (s.e.)			
Scenario			All SNPs		Four surrounding SNPs	
Base	HF^1^	HF^1^	0.942	(0.003)	0.766	(0.011)
1	HF^1^	GWH^3^	0.378	(0.018)	0.569	(0.020)
2	HF^1^ + MRY^2^	GWH^3^	0.377	(0.017)	0.579	(0.020)
3	HF^1^	MRY^2^	0.362	(0.018)	0.562	(0.020)
4	HF^1^ + GWH^3^	MRY^2^	0.373	(0.018)	0.567	(0.021)

^1^ *HF* Holstein Friesian

^2^ *MRY* Meuse-Rhine-Yssel

^3^ *GWH* Groninger White Headed

**Fig. 3:**
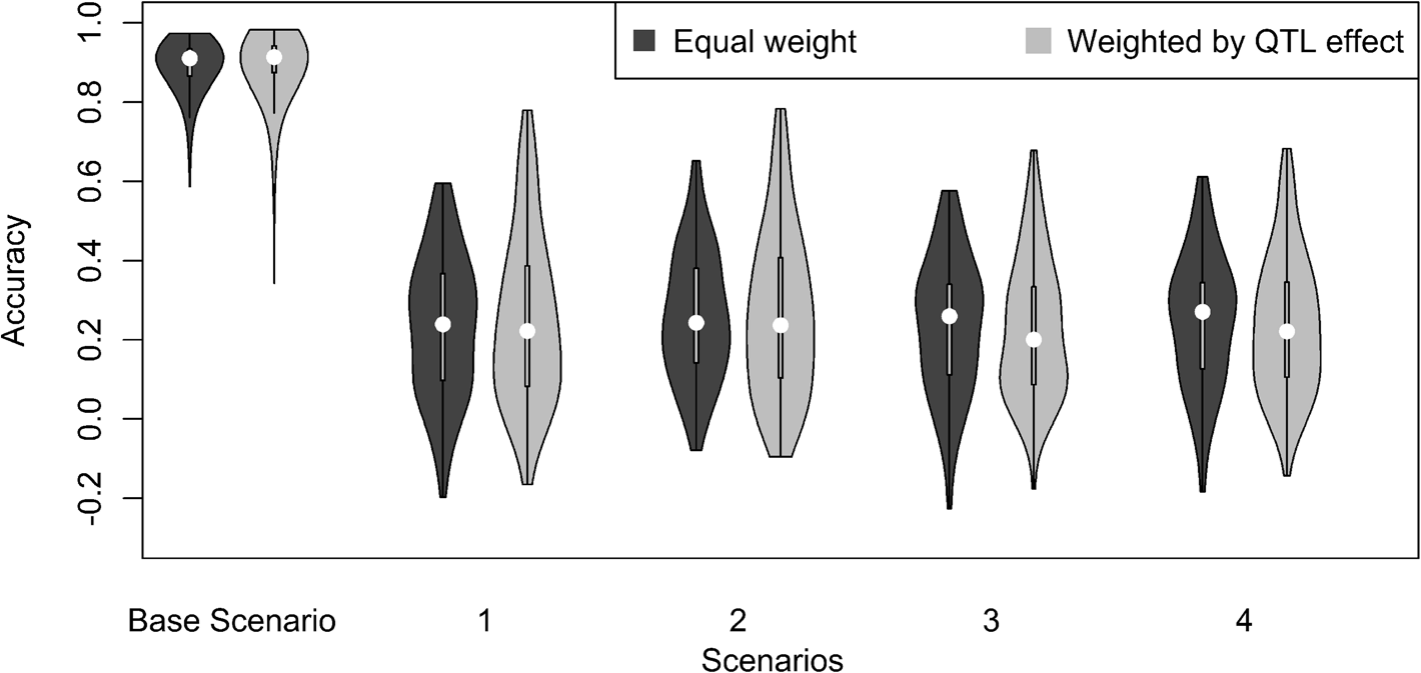
Accuracies of predicing genotypes of QTL with low MAF using selection index theory. Violin plot depicting the accuracies of selection index theory to predict the QTL genotypes of three QTL with low MAF using an equal weight for each of the QTL, or different weights for each QTL, based on their allele substitution effects, in the overall breeding goal for five different scenarios. Base = reference population Holstein Friesian (HF), selection candidates HF; 1 = reference population HF, selection candidates Groninger White Headed (GWH); 2 = reference population HF and Meuse-Rhine-Yssel (MRY), selection candidates GWH; 3 = reference population HF, selection candidates MRY; 4 = reference population HF and GWH, selection candidates MRY

In Fig. 3, the values for acc_MLLD are shown when 3 QTL were underlying the trait and when QTL were sampled with a low MAF. The results show that acc_MLLD was lower for all scenarios when the MAF of the QTL was lower, confirming the expectation that the strength of LD is reduced when the MAF of the QTL is lower. The decrease in acc_MLLD was, however, much lower for the within population scenario where acc_MLLD was around 95 % of the acc_MLLD with QTL randomly sampled, than for the across population scenarios where acc_MLLD was around 60 – 70 % of the acc_MLLD with QTL randomly sampled.

### Accuracy of genomic prediction

**Fig. 4:**
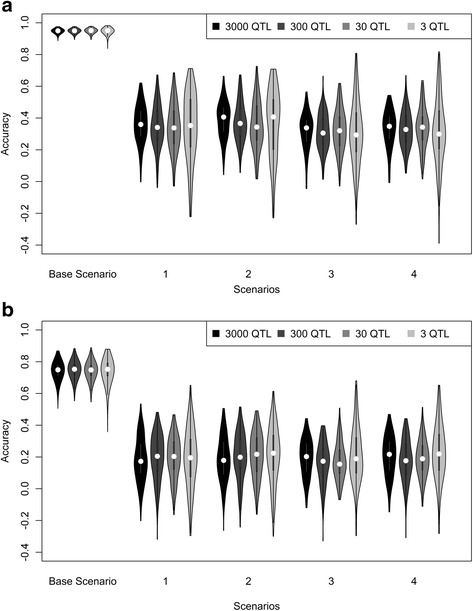
Accuracies of predicting genomic breeding values using GREML for different scenarios using multiple populations. Violin plot depicting the accuracies of genomic prediction using GREML and a (**a**) heritability of 0.95, or (**b**) heritability of 0.3 for five different scenarios. Base = reference population Holstein Friesian (HF), selection candidates HF; 1 = reference population HF, selection candidates Groninger White Headed (GWH); 2 = reference population HF and Meuse-Rhine-Yssel (MRY), selection candidates GWH; 3 = reference population HF, selection candidates MRY; 4 = reference population HF and GWH, selection candidates MRY

Accuracies of predicting genomic estimated breeding values, hereafter denoted as acc_GEBV, achieved with a GREML model are shown in Fig. 4, for a heritability of 0.95 (A) and a heritability of 0.3 (B). At a heritability of 0.95, the average acc_GEBV for the within population scenario was around 0.95, and was much lower and in the range of 0.3 – 0.4 across populations. At a heritability of 0.3, average acc_GEBV was lower for all scenarios, with values around 0.75 for the within population scenario and values around 0.2 for the across population scenarios. For all scenarios, acc_GEBV was independent from the number of QTL underlying the trait and standard errors were reasonably small, although slightly larger for the across population scenarios compared to the within population scenarios.

The acc_GEBV for GWH individuals were somewhat higher (~0.04 at a heritability of 0.95; and ~0.005 at a heritability of 0.3) than predicting MRY individuals using a HF reference population. When the reference population was extended with the other population, acc_GEBV increased slightly, although not significantly, for both populations (~0.015).

Table 4 shows the average acc_GEBV when 3 QTL were underlying the trait with QTL randomly selected and QTL selected to have a low MAF for a heritability of 0.95. Those results show that average acc_GEBV was in all scenarios lower when QTL had a low MAF compared to randomly selected QTL. The accuracies achieved for QTL with a low MAF were 98 % and 65 % of the accuracies for randomly selected QTL for respectively the within and across population scenarios, indicating that the decrease in accuracy was smaller for the within population scenario compared to the across population scenarios.

**Table 4:** Average accuracies (s.e.) of genomic prediction using QTL randomly sampled or QTL with low MAF. The results are for different within and across population scenarios with 3 QTL underlying the trait and a heritability of 0.95

Scenario	Reference population	Selection candidates	Average accuracy of genomic prediction (s.e.)			
			QTL randomly sampled		QTL with low MAF	
Base	HF^1^	HF^1^	0.949	(0.001)	0.932	(0.002)
1	HF^1^	GWH^3^	0.341	(0.021)	0.233	(0.022)
2	HF^1^ + MRY^2^	GWH^3^	0.361	(0.022)	0.246	(0.022)
3	HF^1^	MRY^2^	0.304	(0.020)	0.186	(0.018)
4	HF^1^ + GWH^3^	MRY^2^	0.310	(0.021)	0.189	(0.019)

^1^ *HF* Holstein Friesian

^2^ *MRY* Meuse-Rhine-Yssel

^3^ *GWH* Groninger White Headed

### Accuracy of predicting genomic breeding values (acc_GEBV) versus accuracy of predicting QTL genotypes (acc_MLLD)

**Fig. 5:**
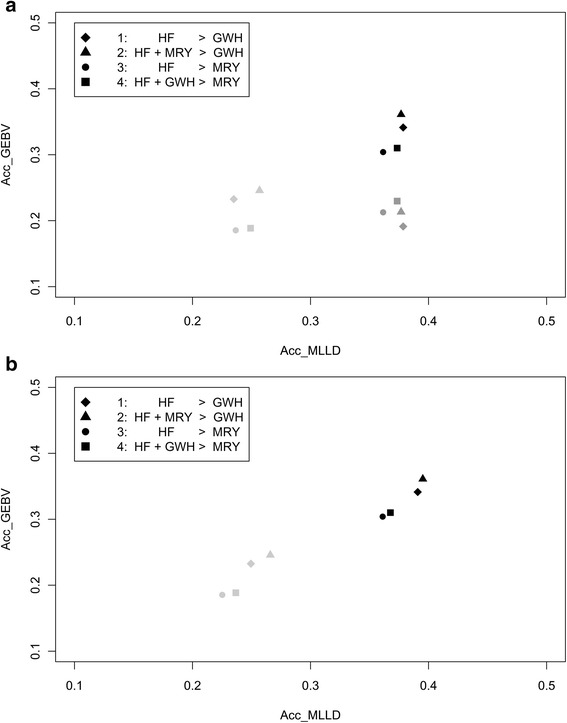
Average accuracies of genomic prediction (Acc_GEBV) versus average accuracies of predicting QTL genotypes (Acc_MLLD) with 3 QTL. Average accuracies of genomic prediction (Acc_GEBV) versus average accuracies of selection index theory to predict the QTL genotypes (Acc_MLLD) with (**a**) equal weight for each of the QTL, or (**b**) QTL weighted based on their allele substitution effects in the overall breeding goal and with 3 QTL underlying the trait randomly sampled using a heritability of 0.95 (black) or 0.3 (dark grey), or QTL selected with a low MAF and a heritability of 0.95 (light grey) for four different scenarios; HF = Holstein Friesian; MRY = Meuse-Rhine-Yssel; GWH = Groninger White Headed

To investigate the relationship between acc_MLLD and acc_GEBV across different across population genomic prediction scenarios, the average acc_GEBV are plotted against the average acc_MLLD in Fig. 5 for the four across population scenarios with 3 QTL underlying the trait. As expected, the average acc_MLLD was for most scenarios equal or higher than the average acc_GEBV. When the heritability was 0.95 and QTL were randomly sampled, the average acc_MLLD was ~0.03 higher than acc_GEBV in the across population scenarios, and the average acc_MLLD and acc_GEBV were similar in the within population scenarios. The differences were larger when the heritability was 0.3 (~0.17 in the across population scenarios, and ~0.20 in the within population scenarios). When QTL were sampled with a low MAF, the differences were comparable to the differences with QTL randomly sampled at a heritability of 0.95 for the across population scenarios. In the within population scenarios, however, the average acc_GEBV was ~0.04 higher than acc_MLLD.

The correlation between acc_GEBV and acc_MLLD was expected to be high and positive, since a high consistency of multi-locus LD across reference individuals and selection candidates is supposed to be very important in getting a high accuracy of genomic prediction. Across the four different across population scenarios and at the same number of randomly sampled QTL underlying the trait and a heritability of 0.95, the average correlation between acc_GEBV and acc_MLLD was 0.91 (range 0.76 to 1.00) when each QTL had an equal weight in the breeding goal, and on average 0.94 (range 0.86 to 1.00) when each QTL had a different weight, based on their different allele substitution effects. When the heritability was only 0.3, the average correlation was lower (0.79). At a heritability of 0.95 and 3 QTL sampled with a low MAF, the correlations were 0.33 and 0.95 when QTL were respectively equally weighted or weighted based on their different allele substitution effects. Altogether, those results show that the measure for consistency of multi-locus LD, acc_MLLD, as calculated in this study using selection index theory, is highly related to the accuracy of genomic prediction obtained with GBLUP.

## Discussion

### Using selection index theory to investigate the consistency of multi-locus LD

The first objective of this study was to investigate the consistency of multi-locus LD across different populations using selection index theory. Our results indicate that the strength of LD reduces when the MAFof the QTL reduces and that LD between QTL and SNPs is at least partly different across populations, especially for loci with a low MAF, resulting in a lower accuracy of predicting the QTL genotypes of selection candidates from another population. When focusing in genomic prediction models only on the SNPs closely located to a QTL, the accuracy of predicting the QTL genotypes of individuals from another population increased, indicating that consistency of LD across populations is higher at shorter distances on the genome. Those findings are in agreement with other studies investigating the consistency of linkage phase between pairs of markers across populations [9, 10], but provide a more complete picture as it considers multi-locus LD. Moreover, the measure for the consistency of multi-locus LD seems to be independent from the number of QTL underlying the trait and the weighting of the QTL in the overall breeding goal of the selection index calculations, but it is depending on the properties of the QTL like allele frequency pattern. Therefore, the consistency of multi-locus LD, as calculated with selection index theory using all SNPs, can be seen as a characteristic of the properties of the QTL for the investigated populations.

### Consistency of multi-locus LD and accuracy of genomic prediction

The second objective of this paper was to investigate the relationship between consistency of multi-locus LD and accuracy of genomic prediction across different within and across population genomic prediction scenarios. As expected, the correlation between average consistency of multi-locus LD and average accuracy of genomic prediction across the different across population scenarios was positive and strong, both at a heritability of 0.95 and 0.3, and when QTL were randomly selected or selected to have a low MAF. The correlations were slightly stronger when QTL were weighted based on their allele substitution effects in the overall breeding goal, since it is more important that the linkage phases between SNPs and QTL with a high effect are consistent across reference and selection individuals compared to QTL with a small effect.

At a heritability of 0.95 and with QTL randomly selected, the correlations between consistency of multi-locus LD and accuracy of genomic prediction were around 0.9. This indicates that around 81 % of the variance in accuracy of genomic prediction could be explained by differences in consistency of multi-locus LD. The remaining part of the variance might be explained by the accuracy of estimating SNP effects, which influenced the accuracy of genomic prediction, but not the consistency of multi-locus LD. The accuracy of estimating SNP effects in the reference population depends on the allele frequency of the QTL, the number of QTL underlying the trait, the heritability of the trait and the size of the reference population [1, 4, 5]. In general, estimated SNP effects are less accurate for traits with a low heritability and for SNPs linked to QTL with a low frequency. This is confirmed by the lower correlations between consistency of multi-locus LD and accuracy of genomic prediction found in this study when the heritability was only 0.3 and when QTL were selected to have a low MAF. The difference in accuracy obtained when QTL were randomly selected compared to selecting QTL with a low MAF was higher for the across population scenarios compared to the within population scenarios. This can be explained by the fact that QTL with a low MAF in the reference population explain only a small part of the genetic variance within the selection candidates when they are from the same population [1]. Due to differences in allele frequencies across populations, the penalty of incorrectly estimating the effects of SNPs linked to QTL with a low MAF might be much higher when selection candidates are from a different population [1]. Combining two or more populations in the reference population might increase the probability that the QTL explaining a large part of the genetic variance in the selection candidates are segregating at reasonable allele frequencies in the reference population. This could explain the slight increase in accuracy of across population genomic prediction when another population was added to the reference population, as seen in this study as well as in other studies [7, 28, 36]. Another explanation for the slight increase in accuracy when combining multiple populations in the reference population could be the assigning of the effect of QTL to SNPs that are more closely located to the QTL [7], for which the consistency of LD across populations is higher [9, 12, 15]. This latter explanation is, however, not confirmed by the values for the consistency of multi-locus LD calculated in this study.

Both the accuracy of predicting the QTL genotype and accuracy of genomic prediction were very high in the single population scenario. Those high values might indicate a strong level of LD within the population, but might also be caused by a high level of family relationships within the population, since family relationships and level of LD are entangled [37]. Both population level LD and LD due to family relationships are helpful in predicting the QTL genotype, resulting in higher accuracies of genomic prediction when the level of family relationships between reference and selection candidates is higher, as was already shown in other studies [2, 38]. Across populations, close family relationships are in general absent, so across population genomic prediction is only depending on the level of LD across the populations, resulting in lower accuracies of genomic prediction. Both the accuracy of predicting the QTL genotype and accuracy of genomic prediction decreased when the MAF of QTL was lower, with a much smaller decrease in the within population scenario compared to the across population scenarios. This might be a result of the possibility to tag QTL with low MAF by the SNPs within a population due to the high level of family relationships. Across populations, it is much more difficult to tag those QTL by the SNPs, since only the level of LD across the populations can be used. This indicates that the effect of the MAF of QTL might be much larger for across population genomic prediction compared to within population genomic prediction.

By focusing only on the four neighboring SNPs of a QTL, the accuracy of predicting the QTL genotype of the selection candidates substantially decreased within a population, but substantially increased in the across population scenarios. This indicates that SNPs further away from the QTL on the genome can be helpful in predicting the QTL genotype within a population, but can be detrimental for across population settings, due to the lower consistency of LD across populations [9, 12, 15,]. The potential of combining populations using the current methods of genomic prediction based on all SNPs would therefore be overestimated by only considering the consistency of LD across populations at short distances on the genome. On the other hand, the results do show that the accuracy of across and multi population genomic prediction could potentially be increased by focusing only on the neighboring SNPs of a QTL, for which the consistency of LD is higher across populations.

Within this study, different numbers of QTL were selected and allele substitution effects were drawn from a normal distribution. The actual distribution of allele substitution effects may perhaps be closer to a gamma distribution [39], showing few QTL with large effects and many QTL with small effects. In such case, the achieved accuracy mainly depends on the ability to tag those few QTL [40], so effectively is rather similar to our simulations with only 3 QTL underlying the trait. Since the number of QTL underlying the trait had no effect on the consistency of multi-locus LD and the accuracy of genomic prediction in the GBLUP model, we expect that the results of our study are also valid when QTL effects follow a gamma distribution.

Altogether, the results of this study show that consistency of multi-locus LD can be used to get more insight in possible underlying reasons and potential ways to increase the low empirical accuracies of across population genomic prediction described in literature, e.g. [16, 36, 41], as follows. When a low accuracy of across population genomic prediction is accompanied by a low consistency of multi-locus LD, a higher marker density might be used to increase the accuracy of genomic prediction. When a low accuracy is not accompanied by a low consistency of multi-locus LD, it indicates that the accuracy of estimating SNP effects is low. This might be caused by differences in allele substitution effects across populations, due to the presence of non-additive effects and differences in allele frequencies across populations [37]. In genetic analyses, those differences can be taken into account by estimating the genetic correlation across the populations [28, 42]. Another reason for the low accuracy of estimating SNP effects might be that the allele frequency of the QTL explaining a large part of the genetic variance in the selection candidates is too low in the reference population, the effect of this might be reduced by including another population in the reference population.

### Potential applications

Our results showed that consistency of multi-locus LD across populations was not influenced by the number of QTL or by the weighting of QTL in the overall breeding goal. This indicates that the consistency of multi-locus LD is not trait-dependent and that, even when the actual QTL are unknown, reliable estimates of the consistency of multi-locus LD can be obtained by sampling loci from the SNPs. The characteristics of the QTL, such as allele frequency, however, influenced the consistency of multi-locus LD and accuracy of genomic prediction. The effect of MAF of QTL on accuracy was already shown in other studies [43, 44], but the results of this study confirm the hypothesis that this effect was due to a reduction in the strength of LD between SNPs and QTL. Therefore, it is highly recommended, assuming that the knowledge about the distribution of allele frequencies of QTL increases in the next decade, to select loci that have comparable allele frequencies as the actual QTL underlying the trait of interest in future applications. Since the main conclusions of this study remain valid when the characteristics of the QTL are taken into account, we expect that those conclusions are also valid for traits with other characteristics, for other breeds and even for other species.

The computational demands for the selection index calculations would be high when including all SNPs on the genome. For practical applications, it might therefore be beneficial to only include a subset of the chromosomes in the analyses which have a representative LD pattern for the whole genome. Computational demands can also be reduced by decreasing the number of QTL, which also reduces the number of potential singularities in the correlation matrices between QTL, since the number of QTL did not have a large impact on the accuracy of predicting the QTL genotype. The number of QTL did, however, influence the variance across the replicates. Therefore, multiple replicates would be necessary when a rather small number of QTL is selected.

## Conclusions

In this paper, selection index theory was used to obtain a measure for the consistency of multi-locus LD across the reference and selection populations. As expected, the consistency of multi-locus LD across populations, when reference and selection candidates were from different populations, was much lower compared to the consistency of multi-locus LD within a population, when reference and selection individuals belonged to the same population. Moreover, the consistency of multi-locus LD was much lower for QTL with a low MAF compared to randomly selected QTL. The average consistency of multi-locus LD is shown to be independent from the number of QTL and the weighting of the QTL in the overall breeding goal of the selection index. Therefore, consistency of multi-locus LD can be seen as a characteristic of the properties of the QTL for the investigated populations. Across different across population scenarios, consistency of multi-locus LD was highly correlated with the achieved accuracy of genomic prediction using a GBLUP type of model, confirming that consistency of LD is an import factor determining the accuracy of across population genomic prediction. Therefore, the consistency of multi-locus LD can provide more insight in underlying reasons for a low empirical accuracy of across population genomic prediction. By focusing only on the SNPs closely located to a QTL, the accuracy of predicting the QTL genotypes of individuals from another population increased. This shows that accuracy of across and multi population genomic prediction could be increased by focusing only on the neighboring SNPs of a QTL, for which the consistency of LD is higher across populations.

## Additional files

Additional file 1: Figure S1. Absolute estimated regression coefficients (b-values) for each SNP to predict the QTL genotypes of 30 randomly selected QTL. Absolute regression coefficients for each of the SNPs estimated in a Holstein Friesian reference population (b_*RP*_) to predict the QTL genotypes of 30 randomly selected QTL with (A) equal weight for each of the QTL, or (B) QTL weighted differently, based on their allele substitution effects, in the overall breeding goal. The size of the triangle represents the weight of the QTL in the overall breeding goal of the selection index calculations, i.e. the allele substitution effect in (B). **Figure S2.** – Absolute estimated regression coefficients (b-values) for each SNP to predict the QTL genotypes of 300 randomly selected QTL. Absolute regression coefficients for each of the SNPs estimated in a Holstein Friesian reference population (b_*RP*_) to predict the QTL genotypes of 300 randomly selected QTL with (A) equal weight for each of the QTL, or (B) QTL weighted differently, based on their allele substitution effects, in the overall breeding goal. The size of the triangle represents the weight of the QTL in the overall breeding goal of the selection index calculations, i.e. the allele substitution effect in (B). **Figure S3.** – Absolute estimated regression coefficients (b-values) for each SNP to predict the QTL genotypes of 3000 randomly selected QTL. Absolute regression coefficients for each of the SNPs estimated in a Holstein Friesian reference population (b_*RP*_) to predict the QTL genotypes of 3000 randomly selected QTL with (A) equal weight for each of the QTL, or (B) QTL weighted differently, based on their allele substitution effects, in the overall breeding goal. The size of the triangle represents the weight of the QTL in the overall breeding goal of the selection index calculations, i.e. the allele substitution effect in (B). Additional file 2: Figure S4. Absolute estimated regression coefficients (b-values) for each SNP to predict the QTL genotypes of 3 QTL with a low MAF. Absolute regression coefficients for each of the SNPs estimated in a Holstein Friesian reference population (b_*RP*_) to predict the QTL genotypes of 3 QTL with a low MAF with (A) equal weight for each of the QTL, or (B) QTL weighted differently, based on their allele substitution effects, in the overall breeding goal. The size of the triangle represents the weight of the QTL in the overall breeding goal of the selection index calculations, i.e. the allele substitution effect in (B).

## Abbreviations

LD: Linkage disequilibrium
SNP: Single nucleotide polymorphism
QTL: Quantitative trait loci
GREML: Genomic-relationship-matrix residual maximum likelihood
**GRM**: Genomic relationship matrix
GBLUP: Genomic best linear unbiased prediction
REML: Residual Maximum Likelihood
HF: Holstein Friesian
GWH: Groninger White Headed
MRY: Meuse-Rhine-Yssel
BTA: *Bos Taurus* chromosome
TBV: True breeding value
MAF: Minor allele frequency
**A**: Pedigree based relationship matrix
MLLD: Multi-locus linkage disequilibrium
Acc_MLLD: Accuracy of predicting QTL genotypes for selection candidates/consistency of multi-locus linkage disequilibrium
Acc_GEBV: Accuracy of predicting genomic estimated breeding values
