# Supplementary material for: Using selection index theory to estimate consistency of multi-locus linkage disequilibrium across populations
Source: BMC Genet. 2015 Jul 19;16:87. doi: 10.1186/s12863-015-0252-6 (PMC4506610; doi:10.1186/s12863-015-0252-6)
Supplement: Additional file 1: Figure S1. — Absolute estimated regression coefficients (b-values) for each SNP to predict the QTL genotypes of 30 randomly selected QTL. Absolute regression coefficients for each of the SNPs estimated in a Holstein Friesian reference population (bRP) to predict the QTL genotypes of 30 randomly selected QTL with (A) equal weight for each of the QTL, or (B) QTL weighted differently, based on their allele substitution effects, in the overall breeding goal. The size of the triangle represents the weight of the QTL in the overall breeding goal of the selection index calculations, i.e. the allele substitution effect in (B). Figure S2. – Absolute estimated regression coefficients (b-values) for each SNP to predict the QTL genotypes of 300 randomly selected QTL. Absolute regression coefficients for each of the SNPs estimated in a Holstein Friesian reference population (bRP) to predict the QTL genotypes of 300 randomly selected QTL with (A) equal weight for each of the QTL, or (B) QTL weighted differently, based on their allele substitution effects, in the overall breeding goal. The size of the triangle represents the weight of the QTL in the overall breeding goal of the selection index calculations, i.e. the allele substitution effect in (B). Figure S3. – Absolute estimated regression coefficients (b-values) for each SNP to predict the QTL genotypes of 3000 randomly selected QTL. Absolute regression coefficients for each of the SNPs estimated in a Holstein Friesian reference population (bRP) to predict the QTL genotypes of 3000 randomly selected QTL with (A) equal weight for each of the QTL, or (B) QTL weighted differently, based on their allele substitution effects, in the overall breeding goal. The size of the triangle represents the weight of the QTL in the overall breeding goal of the selection index calculations, i.e. the allele substitution effect in (B). [file 12863_2015_252_MOESM1_ESM.pdf]

Additional File 1

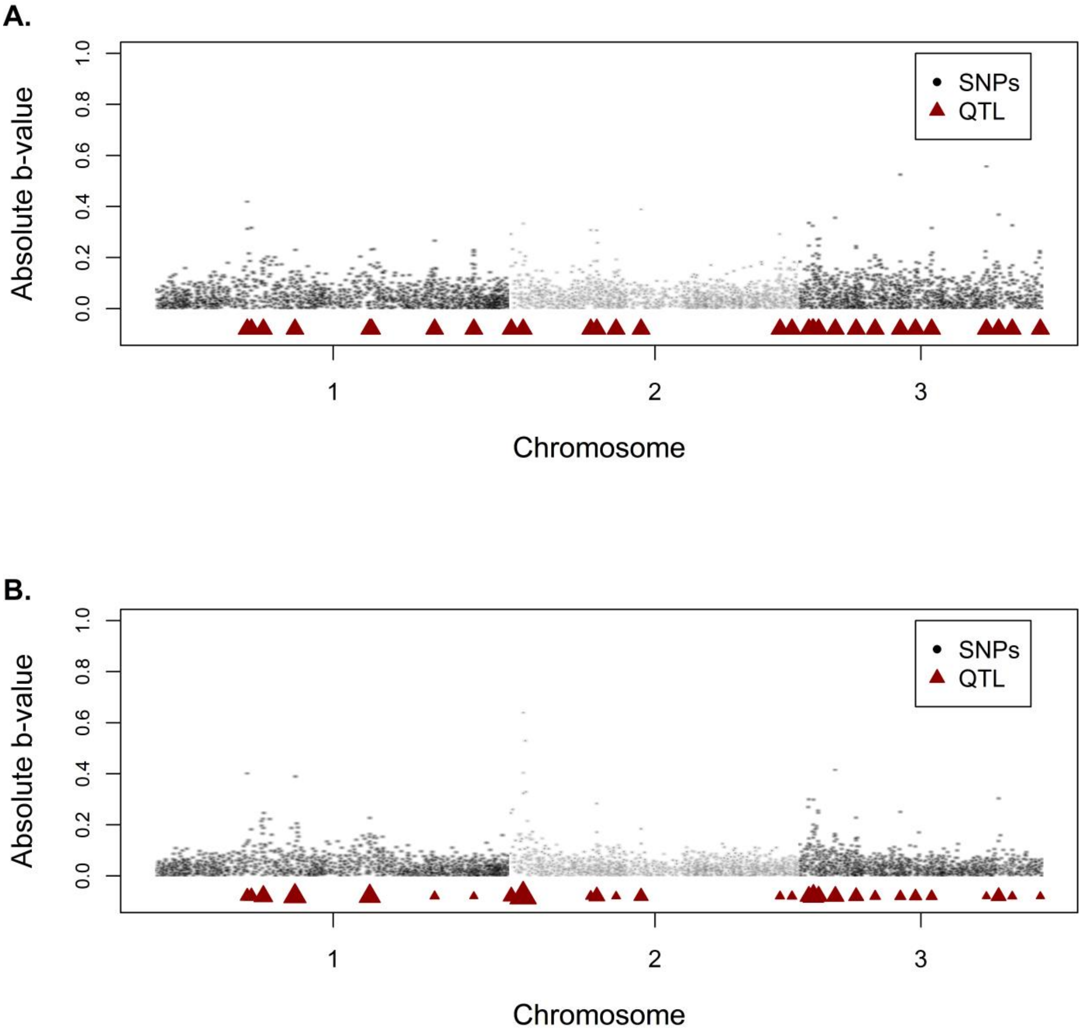

Figure S1 – Absolute estimated regression coefficients (b-values) for each SNP to predict the QTL genotypes of 30 randomly selected QTL.

Absolute regression coefficients for each of the SNPs estimated in a Holstein Friesian reference population ($\mathbf{b}_{RP}$) to predict the QTL genotypes of 30 randomly selected QTL with (A) equal weight for each of the QTL, or (B) QTL weighted differently, based on their allele substitution effects, in the overall breeding goal. The size of the triangle represents the weight of the QTL in the overall breeding goal of the selection index calculations, i.e. the allele substitution effect in (B).

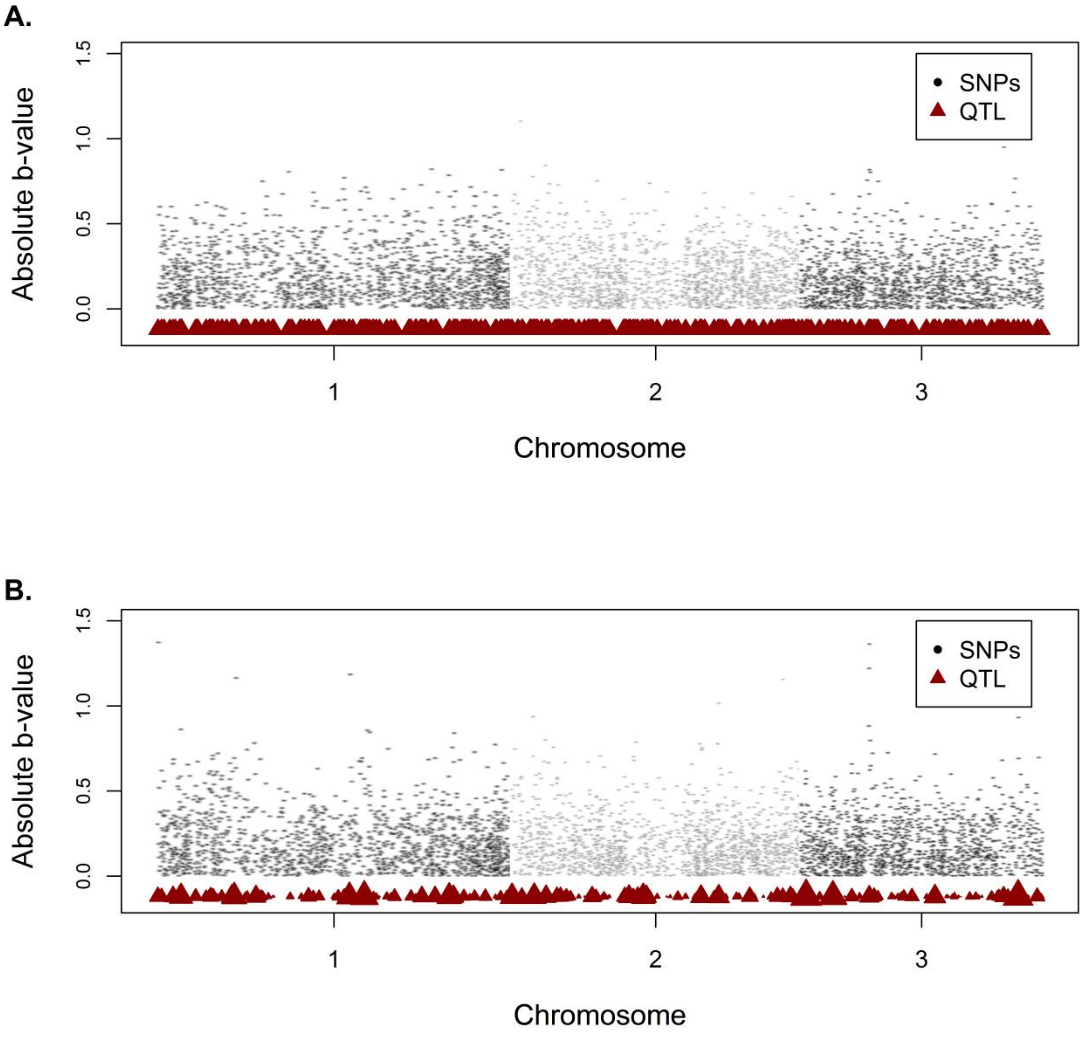

Figure S2 – Absolute estimated regression coefficients (b-values) for each SNP to predict the QTL genotypes of 300 randomly selected QTL.

Absolute regression coefficients for each of the SNPs estimated in a Holstein Friesian reference population ($\mathbf{b}_{RP}$) to predict the QTL genotypes of 300 randomly selected QTL with (A) equal weight for each of the QTL, or (B) QTL weighted differently, based on their allele substitution effects, in the overall breeding goal. The size of the triangle represents the weight of the QTL in the overall breeding goal of the selection index calculations, i.e. the allele substitution effect in (B).

A.

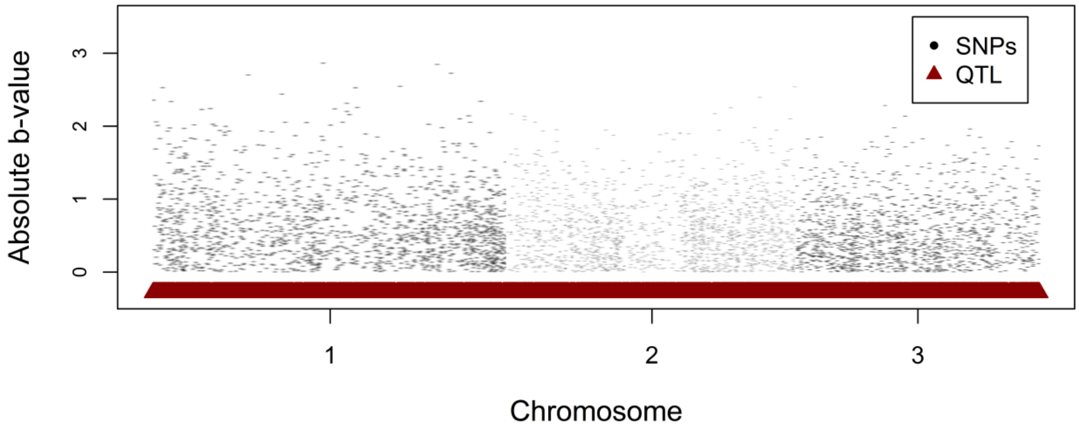

B.

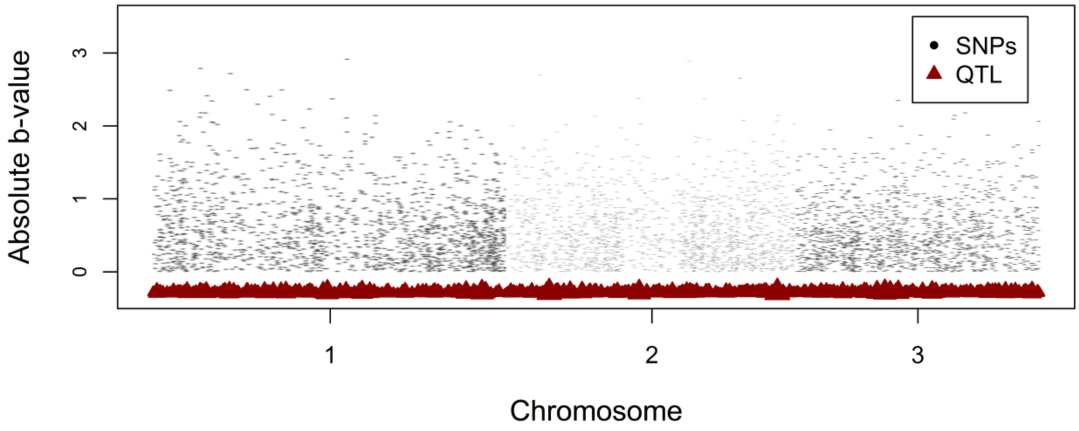

Figure S3 – Absolute estimated regression coefficients (b-values) for each SNP to predict the QTL genotypes of 3000 randomly selected QTL.

Absolute regression coefficients for each of the SNPs estimated in a Holstein Friesian reference population ($\mathbf{b}_{RP}$) to predict the QTL genotypes of 3000 randomly selected QTL with (A) equal weight for each of the QTL, or (B) QTL weighted differently, based on their allele substitution effects, in the overall breeding goal. The size of the triangle represents the weight of the QTL in the overall breeding goal of the selection index calculations, i.e. the allele substitution effect in (B).
